# Proteomic biomarkers in body fluids associated with pancreatic cancer

**DOI:** 10.18632/oncotarget.24654

**Published:** 2018-03-27

**Authors:** Cristina Jimenez-Luna, Carolina Torres, Raul Ortiz, Carmelo Dieguez, Joaquina Martinez-Galan, Consolacion Melguizo, Jose C. Prados, Octavio Caba

**Affiliations:** ^1^ Institute of Biopathology and Regenerative Medicine (IBIMER), Granada University, Granada, Spain; ^2^ Department of Medicine, Division of Gastroenterology and Hepatology, University of Illinois at Chicago, Chicago, IL, USA; ^3^ Department of Health Sciences, Jaen University, Jaen, Spain; ^4^ Department of Gastroenterology, San Cecilio University Hospital, Granada, Spain; ^5^ Department of Oncology, Virgen de Las Nieves University Hospital, Granada, Spain

**Keywords:** proteomics, pancreatic cancer, biomarker, body fluids, diagnosis

## Abstract

Pancreatic cancer (PC) is a highly malignant disease that represents the fourth leading cancer-related death worldwide. There has been very little improvement in survival rates over recent years, and surgical resection remains the only reliable curative approach. Factors that contribute to this dismal prognosis for PC include its rapid progression and invasion, the absence of specific symptoms, and the little impact of available chemotherapy. Importantly, the management of this malignancy is also limited by the lack of highly specific and sensitive biomarkers for its diagnosis and follow-up, and their identification is therefore considered a promising strategy to improve outcomes in these patients. Numerous translational studies have explored the usefulness of body fluids as a non-invasive source of PC-specific biomarkers, and innovations in proteomic methods and technologies have provided a myriad of protein biomarkers for different cancers. The adoption of a proteomic approach has improved understanding of the biology of PC and contributed to the potential identification of protein biomarkers for this disease. This review considers the most recent research efforts to develop novel proteomic biomarkers in body fluids for PC.

## INTRODUCTION

Despite decades of efforts, pancreatic cancer (PC) remains a lethal malignancy with a life expectancy of less than 6 months and a 5-year survival rate of around 5%, due to the rapid progression of the disease and early metastasis [1]. The key symptoms associated with PC are usually unremarkable until the cancer has progressed to an advanced stage, and 80% of patients present with locally advanced or metastatic disease at their diagnosis [2]. Hence, the lack of robust, accurate, and non-invasive methods to detect early stages of PC represents an important obstacle to the improvement of outcomes [3].

The only biomarker routinely used for PC in the clinical setting is serum carbohydrate antigen (CA) 19-9, which is evaluated in the follow-up of already diagnosed patients [4]. However, CA19-9 has inadequate sensitivity (∼80%) and specificity (80-90%) to be useful for PC diagnosis [5], and elevated serum concentrations can also be observed in patients with pancreatitis, benign diseases of the hepatobiliary system, or other malignancies of the gastrointestinal tract [6].

Proteomics, i.e., the large-scale study of proteins, is emerging as a powerful technology to assist in the identification of suitable biomarkers of clinical relevance in PC [7]. Historically, the most widely adopted proteomic technique has been two-dimensional electrophoresis (2DE), which offers high resolution for separating proteins within complex protein mixtures [8]. The two-dimensional difference gel electrophoresis (2D-DIGE) variant allows direct comparison of two protein samples on the same gel, combining conventional 2DE with the sensitivity of fluorescent labeling [9, 10]. Other protein separation methods used in proteomic studies include gel-free approaches such as liquid chromatography (LC), which offers high sensitivity and yields both quantitative and structural information [11]. Protein separation is followed by an identification phase, commonly using mass spectrometry (MS) [10], which plays a key role in this type of study [9]. More advanced MS techniques, such as surface-enhanced laser desorption/ionization (SELDI-MS) and matrix-assisted laser desorption/ionization (MALDITOF-MS), allow protein profiles to be obtained, but their routine clinical application is hampered by the lack of standardized protocols [12]. Other available techniques include protein or antibody microarrays, which provide high resolution to investigate complex proteomes, and multiplexed enzyme-linked immunosorbent assay (ELISA) [13], whose clinical applicability is favored by the small number of target molecules [14]; however, these techniques depend on the availability of well-characterized antibodies [13, 15].

**Figure 1:**
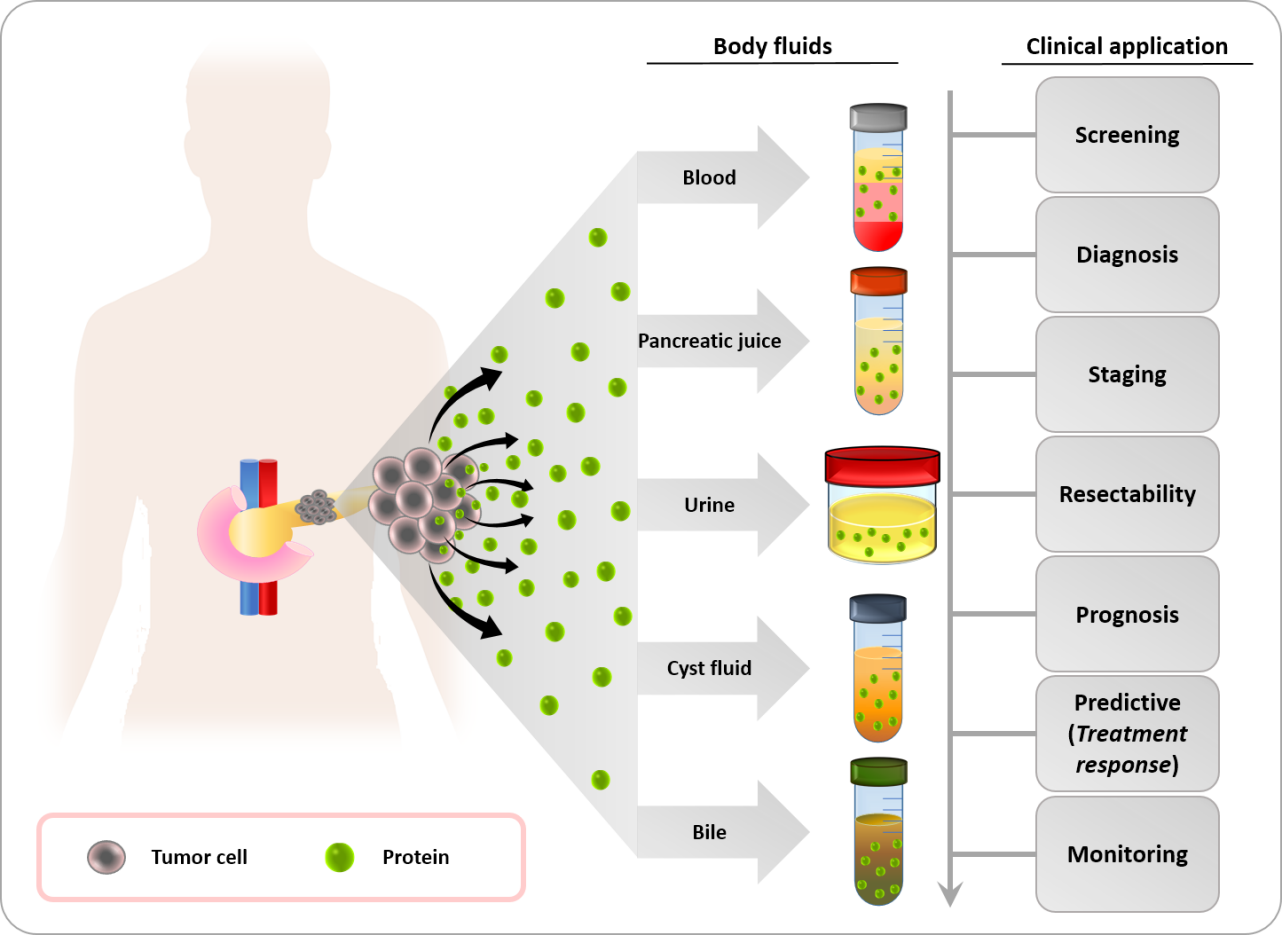
Body fluids for the identification of potential protein biomarkers for pancreatic cancer Blood, pancreatic juice, urine, pancreatic cyst fluid and bile are body fluids that contain cancer-derived proteins. These proteins have a high potential as tumor biomarkers and a number of clinical applications for the management of pancreatic cancer patients, such as screening in high-risk populations for pancreatic cancer, early diagnosis, staging of the disease, assessment of tumor resectability and prognosis, prediction of therapy response to guide treatment decisions, and real-time monitoring of patients.

There has also been a call for more precise and less invasive biomarkers from body fluids to encourage their utilization by clinicians and improve the compliance of PC patients [16]. As shown in Figure 1, there is a broad spectrum of body fluids enriched with proteins that can be used as potential biomarkers for this lethal disease. The purpose of this review was to describe and discuss key findings published over the past three years in relation to proteome-based biomarkers in body fluids for the detection and evaluation of PC.

### Proteomic biomarkers of pancreatic cancer in plasma and serum

Utilization of peripheral blood as a source of circulating proteins for the study of tumor biomarkers is minimally invasive, inexpensive, and highly reproducible [17]. Major efforts have been made to identify new biomarkers for PC in serum or plasma, although this task is complicated by the large number of proteins in blood [18]. Table 1 lists the most recent serum/plasma protein biomarkers proposed for PC.

**Table 1:** Summary of plasma/serum proteomic biomarkers proposed for the management of pancreatic cancer

Single biomarker or panel	Utility	Expression pattern^a^	Impact in prognosis	Year	Ref.
IL-6, IL-8, CEA, or HIF-1α	Prognostic	All ↑	Negative	2016	[19]
IL-6, IL-8, CEA, PDFGFR α, or MUC-1	Prognosis	All ↑	Negative	2016	[19]
HER2	Predictive for erlotinib	↑	Positive	2016	[19]
IL-11	Diagnostic/prognostic	↑/↑	Positive	2014	[20]
IGF-1R	TNM stage	↑	Negative	2014	[21]
EPHB3, IL10, IMPDH2, FGF1, ID1, IL2, SELL, and VCAM1	Diagnostic	↑, ↑, ↑, ↓, ↓, ↓, ↓, and ↓	-	2017	[14]
ICAM 1	Diagnostic	↑	-	2016	[23]
sCD40L	Diagnostic and prognostic/predictive for FOLFIRINOX or GEM+nabpaclitaxel	↑ and ↑/↑	Negative/negative	2014/2016	[25]/[29]
TGF-b1	Prognostic	↑	Negative	2016	[32]
LRG1	Diagnostic/prognostic	↑/↑	Negative	2015	[35]
CRP	Prognostic	↑/↑	Negative/negative	2016/2014	[37]/[38]
Ferritin	Prognostic	↑	Negative	2014	[38]
C4BPA	Diagnostic	↑	-	2016	[39]
HMGB1	Prognostic	↑	Negative	2016	[44]
CD80, PK1, IL-29, NRG1-B1, and PDECGF	Prognostic	All ↑	Negative	2015	[49]
MIC-1	Diagnostic	↑	-	2014	[50]
MIC-1 and ULBP2	Diagnostic	Both ↑	-	2014	[51]
Cofilin-1	Diagnostic/prognostic	↑/↑	Negative	2017	[12]
sgC1qP	Diagnostic/prognostic	↑/↑	Negative	2015	[61]
PRSS2	Diagnostic	↑	-	2015	[62]
DKK1	Diagnostic/prognostic	↑/↑	Negative	2015	[69]
Survivin	Prognostic	↑/↑	Negative/negative	2014/2015	[73]/[74]
LDH	Prognostic/predictive for sorafenib	↑/↓	Negative/positive	2015	[77]
THBS-1	Diagnostic/prognosis	↓/↓	Negative	2016	[78]
THBS-2 and CA19-9	Diagnostic	Both ↑	-	2017	[79]
Exosomal protein (CD44v6, Tspan8, EpCAM, MET, CD104) and miRNA (miR-1246, miR-4644, miR-3976, miR-4306)	Diagnostic	All ↑	-	2015	[82]
Exosomal GPC1	Diagnostic/prognostic	↑/↑	Negative	2015	[83]
CA19-9 and CA242	Diagnostic	Both ↑	-	2015	[84]
CA19-9, CEA, CA125, and CA242	Diagnostic	All ↑	-	2015	[85]
CA125, CA19-9, and LAMC2	Diagnostic	All ↑	-	2014	[86]
Prx-1 and CA 19-9	Diagnostic/prognostic	Both ↑/both ↑	Negative	2015	[87]
CA19-9, IGF1, and albumin	Diagnostic	↑, ↑, and ↓	-	2016	[88]
CA19-9 and MUC-5AC	Diagnostic	Both ↑	-	2017	[89]
CA19-9, TFPI, and TNC-FNIII-B	Diagnostic	↑, ↑, and ↑	-	2017	[90]
TIMP-1, LRG1, and CA19-9	Diagnostic	All ↑	-	2017	[91]
CA19-9, TIMP1, and Apo-AIV	Diagnostic	↑, ↑, and ↓	-	2017	[15]
CA19-9, IGFBP2, and IGFBP3	Diagnostic	↑, ↑, and ↓	-	2016	[92]
C5, IGFBP2, LDHB, PPBP, IGFBP3, and CPN2	Diagnostic	↑, ↑, ↑, ↑, ↓, and ↓	-	2016	[93]
CA19-9, CEA, HGF, OPN, and ctDNA (*KRAS* mutations)	Diagnosis/prognosis	All ↑/all ↑	Negative	2017	[95]
Apo-AI and TF	Diagnostic/prognosis *(only TF)*	Both ↓/both ↓	Negative	2016	[97]
Apo-AII-ATQ/AT and CA19-9	Diagnostic	↓ and ↑	-	2015	[99]
IP-10, IL-6, PDGF, and CA19-9	Diagnostic	All ↑	-	2014	[45]
IL-8, CA19-9, IL-6, and IP-10	Diagnostic	All ↑	-	2014	[45]
IP-10, IL-8, IL-1b, and PDGF	Diagnostic	All ↑	-	2014	[45]
MMP-7 and MMP-12	Diagnostic	Both ↑	-	2014	[46]
Osteoprotegerin	Diagnostic	↑	-	2014	[47]
FGF-10, CXCL11, OSM, GPNMB, and SCF	Diagnostic	All ↑	-	2014	[48]
TNFSF8, CHRDL2, FGF-10, GDF-15, CXCL11, OSM, and SCF	Predictive	↓, ↓, ↑, ↓, ↑, ↑ and ↓	Negative	2014	[48]

^a^ Expression pattern observed in pancreatic cancer patients. ↑: upregulated. ↓ downregulated. GEM: gemcitabina.

#### Single and multiple biomarkers not including CA19-9

Phase III trial PA.3 recently reported that several plasma protein-based biomarkers were associated with tumor stage and survival in PC; thus, interleukin (IL)-6, IL-8, carcinoembryonic antigen (CEA), and hypoxia-inducible factor 1-alpha (HIF-1α) levels were related to the prognosis, while increased IL-8, CEA, platelet-derived growth factor receptor alpha (PDFGFRα), and mucin (MUC)-1 concentrations were related to metastasization. It was also observed that the overall survival (OS) of patients with elevated receptor tyrosine-protein kinase erbB-2 (HER2) concentrations was improved by treatment with erlotinib (*vs*. placebo), suggesting a possible role for HER2 as a predictor of the response to this drug [19]. Plasma IL-11 concentrations have also been proposed as a diagnostic, predictive, and prognostic biomarker for PC, with a sensitivity of 97.7% and specificity of 70% [20]. A correlation was also described between plasma insulin-like growth factor 1 receptor (IGF-1R) expression and TNM stage in PC patients, although no significant difference was found between PC patients and healthy controls and no association was observed between survival and this expression [21]. In 2017, Mustafa et al. [14] proposed a biomarker panel for PC diagnosis comprising eight proteins related to cell adhesion, migration, proliferation, and immunity, among others; this panel demonstrated an accuracy of 95% to discriminate between PC patients and healthy individuals but was unable to differentiate between PC and chronic pancreatitis.

In a study of 66 patients with PC, 43 patients with chronic pancreatitis, and 104 healthy controls, Gebauer et al. [22] found that serum epithelial cell adhesion molecule (EPCAM) concentrations, measured by ELISA, offered only low sensitivity (66.7%) and specificity (77.5%) for PC diagnosis. Other authors found that PC and non-cancer cases could be differentiated using serum concentrations of intercellular adhesion molecule 1 (ICAM1), reporting higher sensitivity and specificity for this biomarker than for CA19-9, although it was not possible to discriminate between early and late tumor stages [23]. It should also be taken into account that serum concentrations of ICAM1 and also of tissue inhibitor of metalloproteinase 1 (TIMP1) can be increased by biliary obstruction in cancer patients, with observations of a lower increase in these concentrations in PC patients with *versus* without biliary obstruction [24].

Over the past few decades, advances in immunotherapy have focused attention on immune signaling pathways in the search for cancer biomarkers. In this regard, the soluble form of CD40 ligand (sCD40L), whose serum concentrations have demonstrated prognostic value in PC patients [25], has been implicated in inflammation, angiogenesis, and immune suppression, among other tumor processes, *via* its CD40 receptor [26–28]. There has been a recent report on the potential of this molecule as a predictive biomarker in metastatic PC patients treated with FOLFIRINOX or gemcitabine plus nab-paclitaxel, observing increased sCD40L concentrations in patients with progressive disease and reduced concentrations in those with a partial response to three months of treatment [29]. CD40 has also been related to transforming growth factor beta 1 (TGF-β1) [28], another key protein implicated in angiogenesis, immune suppression, and cell migration [30, 31] and proposed as a prognostic biomarker for PC. Thus, Zhao et al. [32] found higher serum TGF-β1 concentrations in patients with PC than in patients with benign pancreatic disease or healthy controls and observed a correlation of this increase with more advanced tumor stage and metastasis.

Leucine-rich alpha-2-glycoprotein 1 (LRG1), initially identified as a serum inflammatory protein [33], was recently related to angiogenesis *via* the modulation of endothelial TGF-β1 signaling [34]. In another study, higher serum LRG1 concentrations were observed in patients with PC than in those with chronic pancreatitis or healthy individuals and were also associated with disease progression and lymph node metastasis [35].

The association between inflammation and cancer is well documented [36], and serum C-reactive protein (CRP) concentrations have been correlated with the aggressiveness of PC [37]. A phase III clinical trial in 159 unresectable PC patients described a correlation between increased serum ferritin or CRP concentrations and lower OS, although no correlation was found between these biomarkers [38]. Serum concentrations of complement component 4 binding protein alpha (C4BPA) were reported to be altered in patients with PC and other gastroenterological cancers and to allow the detection of early-stage PC and differentiation between PC and other gastrointestinal cancers [39]. Interestingly, C4BPA has binding sites for numerous ligands, including CD40 or CRP, suggesting its implication in inflammatory processes [40, 41].

High-mobility group box 1 (HMGB1) has also been proposed as a biomarker for PC. It is involved in multiple signaling pathways (e.g., inflammation, immunity, proliferation, metastasis, and apoptosis), and appears to play contradictory roles in cancer according to its cellular localization [42, 43]. Thus, Wu et al. [44] related serum HMGB1 overexpression to shorter OS and progression-free survival in several types of cancer, including PC, whereas other authors proposed a tumor suppressor role for intracellular HMGB1 [43].

Various groups have investigated cytokines as candidate biomarkers for PC due to their implication in numerous cancer-related processes, such as the immune response, inflammation, and metastasis [45–48]. Torres et al. [49] proposed a prognostic biomarker panel for PC comprising five serum cytokines, including CD80, prokineticin 1 (PK1), IL-29, neuregulin 1 (NRG1-beta1), and thymidine phosphorylase (PDECGF), based on the association between their serum concentrations and a poor prognosis. In a study with 1472 participants, serum macrophage inhibitory cytokine 1 (MIC-1) concentrations were found to be increased in the majority of patients with PC, even in those with early-stage disease and negative for CA19-9, suggesting a role for MIC-1 as a biomarker for PC diagnosis and follow-up [50]. In addition, the detection in serum of MIC-1 in combination with UL16 binding protein 2 (ULBP2) was found to improve the accuracy of differentiation between patients with PC and patients with chronic pancreatitis or healthy individuals [51].

Given the high metastatic and invasive potential of PC [52–54], research on proteins implicated in these cell processes is of particular interest. Satoh et al. [12] found that serum concentrations of cofilin-1, involved in actin filament dynamics [55] and metastasization [56], were higher in patients with PC than in patients with pancreatitis or healthy individuals; in addition, an association was found between elevated concentrations and a poor prognosis after surgery. The soluble form of the receptor for the globular heads of C1q (sgC1qP) has been linked to inflammation [57, 58] and has also proven to be a key regulator of cell proliferation, adhesion, migration, and invasion [59, 60]. Higher concentrations of this protein were detected, using sandwich ELISA, in serum and malignant pleural (*n* = 23) and peritoneal (*n* = 27) effusions from metastatic PC patients (*n* = 34) than in healthy controls (*n* = 20) [61]. The same study found an increase in serum sgC1qR concentrations during disease progression in ∼70% of serum samples from the PC patients, in parallel with changes in the tumor biomarkers CEA and CA19-9. In a recent study of 23 patients with PC, 30 patients with pancreatitis, and 35 healthy individuals, a correlation was observed between increased serum concentrations of trypsinogen (PRSS) 2 levels and the presence of PC or pancreatitis, suggesting its possible usefulness as diagnostic biomarker for PC [62].

The tumor microenvironment plays a crucial role in PC progression and contributes to metastasis and therapeutic resistance [63]. In this regard, various groups have reported that dickkopf-1 (DKK1), a soluble inhibitor of Wnt/β-catenin signaling that supports an immunosuppressive environment and epithelial-mesenchymal transition (EMT) [64, 65], is frequently overexpressed in cancer [66–68]. The potential usefulness of this protein as diagnostic and prognostic biomarker for PC was demonstrated by Han et al. [69] in a study of 62 patients with early-stage PC (I/II), 78 with advanced PC (stages III/IV), and 92 PC-free individuals, including healthy individuals and patients with benign pancreatic tumor or chronic pancreatitis. Serum DKK1 concentrations were found to be higher in patients with PC, even in early stages, than in cancer-free individuals, and proved more accurate than CA19-9 for PC diagnosis. Less favorable OS rates were also observed in patients with higher serum DKK1 concentrations.

Survivin, an inhibitor of apoptosis has been implicated in antitumor immunity and EMT [70, 71] and proposed as a prognostic indicator in PC [72]. A study of 80 patients with PC and 80 healthy controls found a correlation between increased survivin concentration (by ELISA) and poor OS [73], supported by more recent observations of higher serum survivin concentrations before treatment in patients with PC than in healthy controls and their association with worse outcomes [74].

Hypoxia is frequently observed in the tumor microenvironment of PC, enhancing cell migration and invasion [75]. Biological links among the enzyme lactate dehydrogenase (LDH), hypoxia, and tumor angiogenesis are well established, and LDH concentrations have also been related to resistance to tyrosine kinase inhibitors (TKIs) such as sorafenib [76]. Thus, better progression-free survival (7.6 vs. 2.8 months) and OS (12.7 vs. 5.9 months) outcomes were recorded in sorafenib-treated patients with PC who had lower *versus* higher serum LDH concentrations, indicating the potential usefulness of this enzyme as a prognostic and predictive indicator in this sub-population of patients. In contrast, lower serum LDH concentrations were related to better progression-free survival (3.3 months vs. 2.2 months in patients with high serum LDH concentrations) and OS (8.6 months vs. 5.2 months) [77].

A large-scale study by Jenkinson et al. [78] on the role of the anti-angiogenic molecule thrombospondin (THBS)-1 as diagnostic biomarker for PC analyzed serum samples from patients with PC, chronic pancreatitis, or type 2 diabetes mellitus and from healthy controls and, of particular interest, from PC patients up to 4 years before their diagnosis; results obtained showed a reduction in THBS-1 concentrations up to 24 months before the clinical diagnosis of PC. In contrast, a recent study by Kim et al. [79] found that elevated plasma THBS-2 and CA19-9 concentrations discriminated patients with PC from healthy subjects with 87% sensitivity and 98% specificity. The authors suggested that this discrepancy may result from differences in the history of diabetes between study populations.

A further approach of interest in the proteomic biomarker field is related to exosomes, small membrane vesicles that are secreted by most cell types and are present in the blood and other body fluids [80, 81]. They play a key role in intercellular communications, and their potential usefulness in PC detection has recently been proposed [81]. Thus, Madhavan et al. [82] employed a biomarker panel to detect five proteins (CD44 antigen variant 6 [CD44v6], tetraspanin-8 [Tspan8], EpCAM, proto-oncogene MET [MET], and CD104 antigen [CD104]) and four microRNAs (miR) (miR-1246, miR-4644, miR-3976 and miR-4306) in circulating exosomes. It achieved 93% specificity to discriminate between PC patients and cancer-free groups (patients with benign pancreatic tumor or chronic pancreatitis and healthy individuals). Strikingly, Melo et al. [83] reported 100% specificity and sensitivity values to distinguish patients with early and late stage PC from cancer-free controls (patients with benign pancreas disease and healthy individuals) based on the detection in circulating exosomes of a single protein molecule, cell surface proteoglycan glypican-1 (GPC1). Although the number of PC precursor lesions studied was limited, the optimal accuracy achieved identifies exosomal GPC1 as a promising biomarker for early PC detection.

Given the low diagnostic usefulness of currently available clinical biomarkers for PC, research efforts have been directed towards the identification of new proteomic biomarkers, with considerable success. However, there is a need to verify the most promising findings in large cohorts and to accelerate their routine clinical application, when justified.

#### Biomarker panels including CA19-9

CA19-9 remains the only FDA-approved PC biomarker and has been included in various biomarker panels in order to complement or surpass its accuracy. Thus, a meta-analysis involving 3497 individuals reported a sensitivity of 89% and specificity of 75% for the combination of CA19-9 and CA242, better than the diagnostic performance of each biomarker individually [84]. In another study in 2015, an accuracy of 92.4% (sensitivity of 90.4% and specificity of 93.8%) was obtained for the combination of serum CA19-9, CEA, CA125, and CA242 concentrations [85]. In the same line, Chan et al. [86] reported a better performance for a biomarker panel consisting of CA125, CA19-9, and laminin subunit gamma 2 (LAMC2) than for CA19-9 alone in the detection of PC and even in the differentiation between early-stage PC and chronic pancreatitis. Other authors found that serum peroxiredoxin-1 (Prx-1) concentrations were higher in patients with PC patients than in healthy controls and that their diagnostic value was higher when used in combination with serum CA19-9 than when each biomarker was considered individually [87]. In 2016, Ferri et al. [88] obtained a sensitivity of 93.6% and specificity of 95% for the combination of CA19-9, insulin-like growth factor 1 (IGF1), and albumin, surpassing the diagnostic value of CA19-9 alone to differentiate between PC and chronic pancreatitis. Likewise, a multicenter study of serum samples from different study groups (early-and late-stage PC, chronic pancreatitis, benign pancreatic disease, and healthy controls) by Kaur et al. [89] found that the diagnostic accuracy of CA19-9 was improved by its combination with MUC-5AC, which achieved 83% sensitivity and 75% specificity to discriminate early-stage PC and cancer-free groups and 83% sensitivity and 83% specificity to discriminate between PC and cancer-free groups.

Balasenthil et al. [90] recently tested the accuracy of a biomarker panel comprising plasma CA19-9, tissue factor pathway inhibitor (TFPI), and an isoform of tenascin C (TNC-FNIII-B) to distinguish early-stage PC from different diseases. They carried out multiple blinded validations in samples from patients with early-stage PC (I/II), chronic pancreatitis, or acute biliary obstruction and from healthy individuals. The accuracy was better with the panel (82%) than with CA19-9 alone (69%) in all early-stage PC cohorts, especially in patients with no history of diabetes or pancreatitis. In this regard, Capello et al. [91] demonstrated that the combination of TIMP1, LRG1, and CA19-9 improved differentiation between patients with early-stage PC and those with benign pancreatic disease or healthy controls in comparison to CA19-9 alone.

Another panel proposed for PC detection was based on serum CA19-9, apolipoprotein (Apo)-AIV, and TIMP1 and obtained a sensitivity of 86% and specificity of 90% in the differentiation of early-stage PC from pancreatitis, higher values than achieved using CA19-9 alone (71% and 90%, respectively) [15]. Yoneyama et al. [92] observed that the diagnostic value of CA19-9 to detect early-stage PC was improved when combined with insulin-like growth factor binding protein (IGFBP) 2 and IGFBP3. Likewise, Kim et al. [93] recently reported that a biomarker panel comprised of six proteins, including IGFBP2 and IGFBP3, had a high capacity to distinguish patients with intraductal papillary mucinous neoplasm (IPMN) from controls (healthy individuals or with other benign disease).

An alternative approach to biomarker discovery is the study of combinations of different types of molecule, including genes, proteins, and RNAs, among others, given that all affect the development of cancer [13, 94]. A recent study of plasma samples from 221 patients with resectable PC and 182 healthy controls tested combinations of genomic and proteomic biomarkers [95]; the authors reported sensitivity of >60% and specificity of ∼100% for early PC detection using a combination of circulating tumor DNA (ctDNA) (mutations in KRAS proto-oncogene, GTPase [*KRAS*]), with protein biomarkers (CA19-9, CEA, hepatocyte growth factor [HGF], and osteopontin [OPN]).

Although CA19-9 is the gold standard for the management of PC, this serum antigen is not expressed in over 10% of the general population, who are Lewis-negative [96]. Lin et al. [97] analyzed serum samples from CA19-9-negative (*n* = 34) and -positive (*n* = 44) patients with PC and healthy controls (*n* = 36) and reported the diagnostic usefulness of Apo-AI and transferrin (TF) concentrations as biomarkers in CA19-9-negative PC patients. Another group investigated plasma/serum concentrations of various Apo-AII isoforms in this context and found a significant decrease in Apo-AII-ATQ/AT concentrations in patients with PC compared with healthy controls [98]. The same group recently developed an antibody-based proteomic approach (using ELISA) for clinical applications, which was tested in a large-scale study with 1156 participants, including 151 patients with PC stage I/II [99]. According to the results, a greater accuracy to discriminate patients with early-stage PC from healthy controls and to identify patients at high risk for PC was achieved by using CA19-9 in combination with Apo-AII-ATQ/AT than by using CA19-9 alone.

The inclusion of CA19-9 in biomarker panels is appealing because it is the “gold standard” for PC diagnosis. One potentially valuable clinical approach could be to continue managing the disease using this well-known biomarker but combine it with others that improve its sensitivity and specificity, increasing the accuracy.

### Proteomic biomarkers of pancreatic cancer in urine

A major advantage of urine as source of biomarkers is that it can be obtained non-invasively; however, its separation from the tumor by the circulation and kidneys represents an important limitation [100]. Nevertheless, recently various groups have proposed certain proteins present in urine as biomarkers for PC (Table 2).

**Table 2:** Summary of urinary proteomic biomarkers proposed for the management of pancreatic cancer

Single biomarker or panel	Utility	Expression pattern^a^	Impact in prognosis	Year	Ref.
LYVE-1, REG-1-alpha, and TFF-1	Diagnostic	All ↑	-	2015	[101]
D-dimer	Preoperative resectability	↑	Negative	2014	[102]

^a^ Expression pattern observed in pancreatic cancer patients. ↑: upregulated. ↓ downregulated.

A study of urinary proteomes in samples from patients with PC or chronic pancreatitis and healthy controls selected the following biomarkers as candidates for the detection of early-stage PC: lymphatic vessel endothelial hyaluronan receptor 1 (LYVE-1), regenerating gene (REG)-1-alpha, and trefoil factor (TFF)-1. These biomarkers showed areas under the curve (AUC) between 0.89 and 0.92 (192 samples from PC patients *vs*. 87 from healthy controls) and between 0.90 and 0.93 (71 samples from PC stage I/II patients *vs*. 87 from healthy controls) and were proposed as a new panel of biomarkers for early PC detection in urine samples [101].

Other authors measured concentrations of D-dimers, the final degradation products of cross-linked fibrin, in 64 patients with potentially resectable pancreatic head tumor and without detectable venous thrombosis in order to predict the preoperative resectability of the tumor. They observed higher average D-dimer values in the peripheral and in portal blood of patients with unresectable PC but found no significant differences in their bile and urine samples. They concluded that a tumor in patients with high D-dimer levels that appears resectable by imaging techniques can be considered unresectable due to occult hepatic metastases [102].

Urine is an ideal fluid for diagnostic screening tests, because a large volume is readily available from patients in a wholly non-invasive manner. Further research is therefore warranted on the identification and validation of potential urinary biomarkers of PC.

### Proteomic biomarkers of pancreatic cancer in pancreatic juice

Samples of pancreatic juice are usually collected directly from the pancreatic duct during surgery or by endoscopic retrograde cholangiopancreatography-based suction, an invasive procedure that may have adverse effects [103]. Hence, pancreatic juice is not an ideal matrix for screening; however, its main advantage as a source of potential biomarkers is that is in direct contact with the pancreas and therefore enriched with numerous proteins derived from the tumor [104]. Various researchers have attempted to identify PC biomarkers in pancreatic juice (Table 3).

**Table 3:** Summary of pancreatic juice proteomic biomarkers proposed for the management of pancreatic cancer

Single biomarker or panel	Utility	Expression pattern^a^	Impact in prognosis	Year	Ref.
AMYP, PRSS1, GP2-1, CCDC132, REG-1-Alpha, REG-1-Beta, REG-3-Alpha, and LIPRP2	Diagnostic	↑, ↑, ↑, ↑, ↑, ↑, ↑, and ↓	-	2014	[105]
Mucins and S100A8 or S100A9	Prognostic	All ↑	Negative	2014	[106]
MUC-1	Diagnostic	↑	-	2015	[107]
CPA5, LIPRP1, KLK1, HBD, and TTR	Diagnostic	↓, ↓, ↓, ↑, and ↑	-	2015	[108]

^a^ Expression pattern observed in pancreatic cancer patients. ↑: upregulated. ↓ downregulated.

In an LC-MS/MS study of the proteome of pancreatic ductal fluid from patients with PC, pancreatitis, or IPMN and healthy controls [105], higher concentrations of pancreatic amylase (AMYP), PRSS1, glycoprotein GP2-1, coiled-coil domain-containing protein 132 (CCDC132), REG-1-Alpha, REG-1-Beta, and REG-3-Alpha, and lower concentrations of pancreatic lipase-related protein (LIPRP) 2 were observed in samples from patients with cancer than in those from healthy individuals. Most of the proteins identified were secreted proteins (81%), related to proteolysis (52%) or metabolic processes (29%). However, only 12 samples were assessed for protein identification and quantification in this study (three samples for each diagnosis). Another study, also limited by its small sample size, described high concentrations of mucins and S100A8 or S100A9 inflammatory proteins in ductal fluid as predictors of poor survival and suggested that pancreatic ductal fluid was a promising matrix for the identification of prognostic biomarkers [106].

Matsumoto et al. [107] evaluated MUC-1, a membrane-associated mucin, in pancreatic juice samples from 39 patients with malignant pancreatic mass and 31 patients with benign pancreatic mass in order to study the clinical impact of this biomarker. They observed higher MUC-1 concentrations in patients with PC or intraductal papillary mucinous carcinoma (IPMC) than in those with inflammatory pancreatic lesion or IPMN, reporting sensitivity, specificity, and accuracy values for MUC-1 (with a 16 U/mL cut-off) of 79.5%, 64.5%, and 72.9%, respectively.

Finally, in a study of intraoperative and postoperative pancreatic juice samples, a subgroup of 11 surgically-treated patients (9 with PC and 2 with non-malignant neoplasm) showed a higher expression of the biomarkers carboxypeptidase A5 (CPA5), inactive LIPRP1 and kallikrein 1 (KLK1) in those with non-malignant disease and a higher expression of hemoglobin subunit delta HBD and transthyretin (TTR) in those with PC. Moreover, differential expression of nine proteins was observed between the six patients who underwent neoadjuvant chemotherapy and the five who did not, with five of the proteins (pancreatic triacylglycerol lipase [PNLIP], inactive LIPRP1, LIPRP2, chymotrypsinlike elastase family member 3B [CELA3B], and carboxypeptidase A1 [CPA1]) being related to up-front surgery and the other four (Apo-B, fibronectin [FN1], α-2-HS-glycoprotein [AHSG], and inter-α-trypsin inhibitor heavy chain H1 [ITIH1]) to chemotherapy [108].

Given that pancreatic juice collection is invasive, this body fluid is far from ideal for diagnostic screening in general populations. Nevertheless, it contains a large number of pancreatic proteins, i.e., potential biomarkers, and may therefore prove useful for the prognosis and management of individuals at high risk of PC.

### Proteomic biomarkers of pancreatic cancer in other body fluids

The proteome of body fluids varies substantially depending on the proximity of the fluid to the PC and its physiological nature; therefore, proteins that are candidate PC biomarkers may be enriched in some fluids but not in others [109]. This variability has prompted some research groups to search for potential biomarkers in other less conventional body fluids (Table 4).

**Table 4:** Summary of proteomic biomarkers in body fluids proposed for the management of pancreatic cancer

Single biomarker or panel	Sample	Utility	Expression pattern^a^	Year	Ref.
Mucin	Cyst fluid	Diagnostic	↑	2014	[113]
Mucin	Cyst fluid	Diagnostic	↑	2014	[114]
MUC-5AC and MUC2	Cyst fluid	Diagnostic	Both ↑	2017	[115]
MUC-5AC and PSCA	Cyst fluid	Diagnostic	Both ↑	2017	[115]
AFM, REG-1-A, PIGR, and LCN2	Cyst fluid	Diagnostic	↓, ↑, ↑, and ↑	2015	[111]
sLR11	Bile	Diagnostic	↑	2016	[117]

^a^ Expression pattern observed in pancreatic cancer patients. ↑: upregulated. ↓ downregulated.

There is no risk of the malignant transformation of non-mucinous pancreatic cysts, whereas mucinous cysts have the potential to progress to invasive PC [110]. The diagnostic accuracy of current clinical techniques to distinguish between these types of cyst lesion is not satisfactory [111, 112]; therefore, the identification of biomarkers for this purpose may represent a good strategy to improve OS outcomes in these patients. Streitz et al. [113] used one-dimensional SDS polyacrylamide gel electrophoresis (1D-SDS PAGE) and a dual staining method to analyze cyst fluid mucin in 28 patients and reported high sensitivity and specificity (95% and 100%, respectively) to differentiate between mucinous and non-mucinous pancreatic cysts. Another study on the proteomic profile of 79 patients reported that mucinous cysts were detected with 97.5% accuracy and malignant transformation was predicted with 89.7% accuracy [114]. More recently, the same authors described an increased accuracy of 97% for a proteomic panel consisting of MUC-5AC and MUC-2 to discriminate between premalignant/malignant pancreatic cystic lesions and benign disease, and accuracy values of 96% and 95% for the combination of MUC-5AC with prostate stem-cell antigen (PSCA) to identify high-grade dysplasia/cancer and malignant/severely dysplastic lesions, respectively [115], the results for both panels were superior to those obtained with existing diagnostic tools. In the same line, Park and colleagues [111] recently described a panel of four proteins that discriminated mucinous from non-mucinous cysts with an accuracy of 93%.

The complex composition of bile has hampered the search for PC biomarkers in this fluid, but technological advances in proteomics have now made this feasible. Thus, a study of bile samples from 24 patients by Navaneethan et al. [116] detected the differential abundance of certain proteins among participants with holangiocarcinoma, PC, primary sclerosing cholangitis, and benign disease. They reported increased bile concentrations of 18 proteins, including S100A8 and S100A9, and decreased concentrations of 30 others, including TFF-2, in the patients with malignant disease, and found protein similarity between bile and plasma. More recently, Terai and colleagues [117] proposed bile soluble sortilin related receptor 1 (sLR11) concentrations as a potential biomarker for PC and biliary tract cancer, based on its significantly higher concentrations in the cancer patients than in those with benign disease in their study population of 72 patients.

The collection of pancreatic juice is not risk-free, and Rocker et al. [103] proposed a promising approach to overcome this drawback based on the secretion of juice into the bowel. They studied the effluent of whole-gut lavage performed for colonoscopy and found that most of the proteins in this fluid were of pancreatic origin. Given that colonoscopy is a routine clinical procedure, this novel approach opens up the possibility of a double-screening procedure for pancreatic and colorectal lesions.

## CONCLUSIONS

PC is one of the most lethal tumors, being the fourth most frequent cause of death from cancer worldwide. The possibility of successful treatment is known to increase with earlier diagnosis, but its detection at an early stage remains challenging, and there is a need for more sensitive and specific biomarkers. Despite advances in the identification of useful biomarkers for many types of cancer, the same success has yet to be achieved for PC. However, the development of a proteomic approach offers new hopes for the discovery of novel protein biomarkers for PC that do not require an invasive procedure.

Liquid biopsies have become more common, and numerous authors have studied the clinical relevance of multiple biomarkers in various body fluids. In some studies, conventional biomarkers have been combined with new molecules to increase their usefulness. The large number of genetic alterations that can underlie PC and the high heterogeneity among patients suggest that significantly more valuable results can be obtained with panels of multiple proteins than with a single biomarker.

Proteomic studies have identified proteins with high potential diagnostic and/or prognostic value in PC and have published abundant information on proteome aberrations that could potentially be therapeutic targets. Results have been promising, but their translation from research laboratories to the clinical setting is disappointingly slow. Hence, there is an urgent need for large-scale validation studies and for close collaboration between researchers and clinicians to develop clinical trials that could accelerate the clinical implementation of useful biomarkers.

## References

[1] Siegel RL, Miller KD, Jemal A (2015). Cancer statistics, 2015. CA Cancer J Clin.

[2] Hidalgo M (2010). Pancreatic cancer. N Engl J Med.

[3] Chan A, Diamandis EP, Blasutig IM (2013). Strategies for discovering novel pancreatic cancer biomarkers. J Proteomics.

[4] Ducreux M, Cuhna AS, Caramella C, Hollebecque A, Burtin P, Goere D, Seufferlein T, Haustermans K, Van Laethem JL, Conroy T, Arnold D (2015). Cancer of the pancreas: ESMO Clinical Practice Guidelines for diagnosis, treatment and follow-up. Ann Oncol.

[5] Goonetilleke KS, Siriwardena AK (2007). Systematic review of carbohydrate antigen (CA 19-9) as a biochemical marker in the diagnosis of pancreatic cancer. Eur J Surg Oncol.

[6] Duffy MJ, Sturgeon C, Lamerz R, Haglund C, Holubec VL, Klapdor R, Nicolini A, Topolcan O, Heinemann V (2010). Tumor markers in pancreatic cancer: a European Group on Tumor Markers (EGTM) status report. Ann Oncol.

[7] Sun C, Rosendahl AH, Ansari D, Andersson R (2011). Proteome-based biomarkers in pancreatic cancer. World J Gastroenterol.

[8] Boschetti E, D’Amato A, Candiano G, Righetti PG (2017 Sep 4). Protein biomarkers for early detection of diseases: The decisive contribution of combinatorial peptide ligand libraries. J Proteomics.

[9] Geng R, Li Z, Li S, Gao J (2011). Proteomics in pancreatic cancer research. Int J Proteomics.

[10] Monteoliva L, Albar JP (2004). Differential proteomics: an overview of gel and non-gel based approaches. Brief Funct Genomic Proteomic.

[11] Coleman O, Henry M, McVey G, Clynes M, Moriarty M, Meleady P (2016). Proteomic strategies in the search for novel pancreatic cancer biomarkers and drug targets: recent advances and clinical impact. Expert Rev Proteomics.

[12] Satoh M, Takano S, Sogawa K, Noda K, Yoshitomi H, Ishibashi M, Mogushi K, Takizawa H, Otsuka M, Shimizu H, Miyazaki M, Nomura F (2017). Immune-complex level of cofilin-1 in sera is associated with cancer progression and poor prognosis in pancreatic cancer. Cancer Sci.

[13] Borrebaeck CA (2017). Precision diagnostics: moving towards protein biomarker signatures of clinical utility in cancer. Nat Rev Cancer.

[14] Mustafa S, Pan L, Marzoq A, Fawaz M, Sander L, Ruckert F, Schrenk A, Hartl C, Uhler R, Yildirim A, Strobel O, Hackert T, Giese N (2017). Comparison of the tumor cell secretome and patient sera for an accurate serum-based diagnosis of pancreatic ductal adenocarcinoma. Oncotarget.

[15] Park J, Lee E, Park KJ, Park HD, Kim JW, Woo HI, Lee KH, Lee KT, Lee JK, Park JO, Park YS, Heo JS, Choi SH (2017). Large-scale clinical validation of biomarkers for pancreatic cancer using a mass spectrometry-based proteomics approach. Oncotarget.

[16] Herreros-Villanueva M, Bujanda L (2016). Non-invasive biomarkers in pancreatic cancer diagnosis: what we need versus what we have. Ann Transl Med.

[17] Whitney AR, Diehn M, Popper SJ, Alizadeh AA, Boldrick JC, Relman DA, Brown PO (2003). Individuality and variation in gene expression patterns in human blood. Proc Natl Acad Sci U S A.

[18] Tessitore A, Gaggiano A, Cicciarelli G, Verzella D, Capece D, Fischietti M, Zazzeroni F, Alesse E (2013). Serum biomarkers identification by mass spectrometry in high-mortality tumors. Int J Proteomics.

[19] Shultz DB, Pai J, Chiu W, Ng K, Hellendag MG, Heestand G, Chang DT, Tu D, Moore MJ, Parulekar WR, Koong AC (2016). A Novel Biomarker Panel Examining Response to Gemcitabine with or without Erlotinib for Pancreatic Cancer Therapy in NCIC Clinical Trials Group PA.3. PLoS One.

[20] Ren C, Chen Y, Han C, Fu D, Chen H (2014). Plasma interleukin-11 (IL-11) levels have diagnostic and prognostic roles in patients with pancreatic cancer. Tumour Biol.

[21] Xu JW, Wang TX, You L, Zheng LF, Shu H, Zhang TP, Zhao YP (2014). Insulin-like growth factor 1 receptor (IGF-1R) as a target of MiR-497 and plasma IGF-1R levels associated with TNM stage of pancreatic cancer. PLoS One.

[22] Gebauer F, Struck L, Tachezy M, Vashist Y, Wicklein D, Schumacher U, Izbicki JR, Bockhorn M (2014). Serum EpCAM expression in pancreatic cancer. Anticancer Res.

[23] mohamed A, Saad Y, Saleh D, Elawady R, Eletreby R, Kharalla AS, Badr E (2016). Can Serum ICAM 1 distinguish pancreatic cancer from chronic pancreatitis?. Asian Pac J Cancer Prev.

[24] Jenkinson C, Elliott V, Menon U, Apostolidou S, Fourkala OE, Gentry-Maharaj A, Pereira SP, Jacobs I, Cox TF, Greenhalf W, Timms JF, Sutton R, Neoptolemos JP (2015). Evaluation in pre-diagnosis samples discounts ICAM-1 and TIMP-1 as biomarkers for earlier diagnosis of pancreatic cancer. J Proteomics.

[25] Chung HW, Lim JB (2014). Clinical significance of elevated serum soluble CD40 ligand levels as a diagnostic and prognostic tumor marker for pancreatic ductal adenocarcinoma. J Transl Med.

[26] Elgueta R, Benson MJ, de Vries VC, Wasiuk A, Guo Y, Noelle RJ (2009). Molecular mechanism and function of CD40/CD40L engagement in the immune system. Immunol Rev.

[27] Huang J, Jochems C, Talaie T, Anderson A, Jales A, Tsang KY, Madan RA, Gulley JL, Schlom J (2012). Elevated serum soluble CD40 ligand in cancer patients may play an immunosuppressive role. Blood.

[28] Kim H, Kim Y, Bae S, Kong JM, Choi J, Jang M, Hong JM, Hwang YI, Kang JS, Lee WJ (2015). Direct Interaction of CD40 on Tumor Cells with CD40L on T Cells Increases the Proliferation of Tumor Cells by Enhancing TGF-beta Production and Th17 Differentiation. PLoS One.

[29] Azzariti A, Brunetti O, Porcelli L, Graziano G, Iacobazzi RM, Signorile M, Scarpa A, Lorusso V, Silvestris N (2016). Potential predictive role of chemotherapy-induced changes of soluble CD40 ligand in untreated advanced pancreatic ductal adenocarcinoma. Onco Targets Ther.

[30] Kim JE, Lee KT, Lee JK, Paik SW, Rhee JC, Choi KW (2004). Clinical usefulness of carbohydrate antigen 19-9 as a screening test for pancreatic cancer in an asymptomatic population. J Gastroenterol Hepatol.

[31] Zhao HW, Li YW, Feng R, Yu JB, Li J, Zhang Y, Li JC, Wang YX (2015). TGF-beta/Smad2/3 signal pathway involves in U251 cell proliferation and apoptosis. Gene.

[32] Zhao J, Liang Y, Yin Q, Liu S, Wang Q, Tang Y, Cao C (2016). Clinical and prognostic significance of serum transforming growth factor-beta1 levels in patients with pancreatic ductal adenocarcinoma. Braz J Med Biol Res.

[33] Haupt H, Baudner S (1977). [Isolation and characterization of an unknown, leucine-rich 3.1-S-alpha2-glycoprotein from human serum (author’s transl)]. [Article in German]. Hoppe Seylers Z Physiol Chem.

[34] Wang X, Abraham S, McKenzie JAG, Jeffs N, Swire M, Tripathi VB, Luhmann UFO, Lange CAK, Zhai Z, Arthur HM, Bainbridge J, Moss SE, Greenwood J (2013). LRG1 promotes angiogenesis by modulating endothelial TGF-beta signalling. Nature.

[35] Furukawa K, Kawamoto K, Eguchi H, Tanemura M, Tanida T, Tomimaru Y, Akita H, Hama N, Wada H, Kobayashi S, Nonaka Y, Takamatsu S, Shinzaki S (2015). Clinicopathological Significance of Leucine-Rich alpha2-Glycoprotein-1 in Sera of Patients With Pancreatic Cancer. Pancreas.

[36] Tang F, Wang Y, Hemmings BA, Ruegg C, Xue G (2018). PKB/Akt-dependent regulation of inflammation in cancer. Semin Cancer Biol.

[37] Mitsunaga S, Ikeda M, Shimizu S, Ohno I, Takahashi H, Okuyama H, Ueno H, Morizane C, Kondo S, Sakamoto Y, Okusaka T, Ochiai A (2016). C-Reactive Protein Level Is an Indicator of the Aggressiveness of Advanced Pancreatic Cancer. Pancreas.

[38] Alkhateeb A, Zubritsky L, Kinsman B, Leitzel K, Campbell-Baird C, Ali SM, Connor J, Lipton A (2014). Elevation in multiple serum inflammatory biomarkers predicts survival of pancreatic cancer patients with inoperable disease. J Gastrointest Cancer.

[39] Sogawa K, Takano S, Iida F, Satoh M, Tsuchida S, Kawashima Y, Yoshitomi H, Sanda A, Kodera Y, Takizawa H, Mikata R, Ohtsuka M, Shimizu H (2016). Identification of a novel serum biomarker for pancreatic cancer, C4b-binding protein alpha-chain (C4BPA) by quantitative proteomic analysis using tandem mass tags. Br J Cancer.

[40] Buil A, Tregouet DA, Souto JC, Saut N, Germain M, Rotival M, Tiret L, Cambien F, Lathrop M, Zeller T, Alessi MC, Rodriguez de Cordoba S, Munzel T (2010). C4BPB/C4BPA is a new susceptibility locus for venous thrombosis with unknown protein S-independent mechanism: results from genome-wide association and gene expression analyses followed by case-control studies. Blood.

[41] Brodeur SR, Angelini F, Bacharier LB, Blom AM, Mizoguchi E, Fujiwara H, Plebani A, Notarangelo LD, Dahlback B, Tsitsikov E, Geha RS (2003). C4b-binding protein (C4BP) activates B cells through the CD40 receptor. Immunity.

[42] Kang R, Zhang Q, Zeh HJ, Lotze MT, Tang D (2013). HMGB1 in cancer: good, bad, or both?. Clin Cancer Res.

[43] Kang R, Xie Y, Zhang Q, Hou W, Jiang Q, Zhu S, Liu J, Zeng D, Wang H, Bartlett DL, Billiar TR, Zeh HJ, Lotze MT (2017). Intracellular HMGB1 as a novel tumor suppressor of pancreatic cancer. Cell Res.

[44] Wu T, Zhang W, Yang G, Li H, Chen Q, Song R, Zhao L (2016). HMGB1 overexpression as a prognostic factor for survival in cancer: a meta-analysis and systematic review. Oncotarget.

[45] Shaw VE, Lane B, Jenkinson C, Cox T, Greenhalf W, Halloran CM, Tang J, Sutton R, Neoptolemos JP, Costello E (2014). Serum cytokine biomarker panels for discriminating pancreatic cancer from benign pancreatic disease. Mol Cancer.

[46] Kahlert C, Fiala M, Musso G, Halama N, Keim S, Mazzone M, Lasitschka F, Pecqueux M, Klupp F, Schmidt T, Rahbari N, Scholch S, Pilarsky C (2014). Prognostic impact of a compartment-specific angiogenic marker profile in patients with pancreatic cancer. Oncotarget.

[47] Shi W, Qiu W, Wang W, Zhou X, Zhong X, Tian G, Deng A (2014). Osteoprotegerin is up-regulated in pancreatic cancers and correlates with cancer-associated new-onset diabetes. Biosci Trends.

[48] Torres C, Perales S, Alejandre MJ, Iglesias J, Palomino RJ, Martin M, Caba O, Prados JC, Aranega A, Delgado JR, Irigoyen A, Ortuno FM, Rojas I (2014). Serum cytokine profile in patients with pancreatic cancer. Pancreas.

[49] Torres C, Linares A, Alejandre MJ, Palomino-Morales RJ, Caba O, Prados J, Aranega A, Delgado JR, Irigoyen A, Martinez-Galan J, Ortuno FM, Rojas I, Perales S (2015). Prognosis Relevance of Serum Cytokines in Pancreatic Cancer. Biomed Res Int.

[50] Wang X, Li Y, Tian H, Qi J, Li M, Fu C, Wu F, Wang Y, Cheng D, Zhao W, Zhang C, Wang T, Rao J (2014). Macrophage inhibitory cytokine 1 (MIC-1/GDF15) as a novel diagnostic serum biomarker in pancreatic ductal adenocarcinoma. BMC Cancer.

[51] Zhou YF, Xu LX, Huang LY, Guo F, Zhang F, He XY, Yuan YZ, Yao WY (2014). Combined detection of serum UL16-binding protein 2 and macrophage inhibitory cytokine-1 improves early diagnosis and prognostic prediction of pancreatic cancer. Oncol Lett.

[52] Takahashi H, Sawai H, Funahashi H, Matsuo Y, Yasuda A, Ochi N, Sato M, Okada Y, Takeyama H (2007). Antiproteases in preventing the invasive potential of pancreatic cancer cells. JOP.

[53] Nguyen AV, Nyberg KD, Scott MB, Welsh AM, Nguyen AH, Wu N, Hohlbauch SV, Geisse NA, Gibb EA, Robertson AG, Donahue TR, Rowat AC (2016). Stiffness of pancreatic cancer cells is associated with increased invasive potential. Integr Biol (Camb).

[54] Gharibi A, La Kim S, Molnar J, Brambilla D, Adamian Y, Hoover M, Hong J, Lin J, Wolfenden L, Kelber JA (2017). ITGA1 is a pre-malignant biomarker that promotes therapy resistance and metastatic potential in pancreatic cancer. Sci Rep.

[55] Wang W, Eddy R, Condeelis J (2007). The cofilin pathway in breast cancer invasion and metastasis. Nat Rev Cancer.

[56] Wang Y, Kuramitsu Y, Kitagawa T, Baron B, Yoshino S, Maehara S, Maehara Y, Oka M, Nakamura K (2015). Cofilin-phosphatase slingshot-1L (SSH1L) is over-expressed in pancreatic cancer (PC) and contributes to tumor cell migration. Cancer Lett.

[57] Ghebrehiwet B, Ji Y, Valentino A, Pednekar L, Ramadass M, Habiel D, Kew RR, Hosszu KH, Galanakis DK, Kishore U, Peerschke EI (2014). Soluble gC1qR is an autocrine signal that induces B1R expression on endothelial cells. J Immunol.

[58] Peerschke EI, Ghebrehiwet B (2007). The contribution of gC1qR/p33 in infection and inflammation. Immunobiology.

[59] Kim KB, Yi JS, Nguyen N, Lee JH, Kwon YC, Ahn BY, Cho H, Kim YK, Yoo HJ, Lee JS, Ko YG (2011). Cell-surface receptor for complement component C1q (gC1qR) is a key regulator for lamellipodia formation and cancer metastasis. J Biol Chem.

[60] Feng X, Tonnesen MG, Peerschke EI, Ghebrehiwet B (2002). Cooperation of C1q receptors and integrins in C1q-mediated endothelial cell adhesion and spreading. J Immunol.

[61] Peerschke EI, Brandwijk RJ, Dembitzer FR, Kinoshita Y, Ghebrehiwet B (2015). Soluble gC1qR in Blood and Body Fluids: Examination in a Pancreatic Cancer Patient Cohort. Int J Cancer Res Mol Mech.

[62] Cao J, Xia C, Cui T, Guo H, Li H, Ren Y, Wang S (2015). Correlations between serum trypsinogen-2 and pancreatic cancer. Hepatogastroenterology.

[63] Feig C, Gopinathan A, Neesse A, Chan DS, Cook N, Tuveson DA (2012). The pancreas cancer microenvironment. Clin Cancer Res.

[64] Takahashi N, Fukushima T, Yorita K, Tanaka H, Chijiiwa K, Kataoka H (2010). Dickkopf-1 is overexpressed in human pancreatic ductal adenocarcinoma cells and is involved in invasive growth. Int J Cancer.

[65] D’Amico L, Mahajan S, Capietto AH, Yang Z, Zamani A, Ricci B, Bumpass DB, Meyer M, Su X, Wang-Gillam A, Weilbaecher K, Stewart SA, DeNardo DG (2016). Dickkopf-related protein 1 (Dkk1) regulates the accumulation and function of myeloid derived suppressor cells in cancer. J Exp Med.

[66] Voorzanger-Rousselot N, Goehrig D, Journe F, Doriath V, Body JJ, Clezardin P, Garnero P (2007). Increased Dickkopf-1 expression in breast cancer bone metastases. Br J Cancer.

[67] Tsai JF, Jeng JE, Chuang WL (2012). Dickkopf-1 and hepatocellular carcinoma. Lancet Oncol.

[68] Liu Y, Tang W, Xie L, Wang J, Deng Y, Peng Q, Zhai L, Li S, Qin X (2014). Prognostic significance of dickkopf-1 overexpression in solid tumors: a meta-analysis. Tumour Biol.

[69] Han SX, Zhou X, Sui X, He CC, Cai MJ, Ma JL, Zhang YY, Zhou CY, Ma CX, Varela-Ramirez A, Zhu Q (2015). Serum dickkopf-1 is a novel serological biomarker for the diagnosis and prognosis of pancreatic cancer. Oncotarget.

[70] Tai CJ, Chin-Sheng H, Kuo LJ, Wei PL, Lu HH, Chen HA, Liu TZ, Liu JJ, Liu DZ, Ho YS, Wu CH, Chang YJ (2012). Survivin-mediated cancer cell migration through GRP78 and epithelial-mesenchymal transition (EMT) marker expression in Mahlavu cells. Ann Surg Oncol.

[71] Garg H, Suri P, Gupta JC, Talwar GP, Dubey S (2016). Survivin: a unique target for tumor therapy. Cancer Cell Int.

[72] Deveraux QL, Reed JC (1999). IAP family proteins--suppressors of apoptosis. Genes Dev.

[73] Ren YQ, Zhang HY, Su T, Wang XH, Zhang L (2014). Clinical significance of serum survivin in patients with pancreatic ductal adenocarcinoma. Eur Rev Med Pharmacol Sci.

[74] Dong H, Qian D, Wang Y, Meng L, Chen D, Ji X, Feng W (2015). Survivin expression and serum levels in pancreatic cancer. World J Surg Oncol.

[75] Chen S, Chen JZ, Zhang JQ, Chen HX, Yan ML, Huang L, Tian YF, Chen YL, Wang YD (2016). Hypoxia induces TWIST-activated epithelial-mesenchymal transition and proliferation of pancreatic cancer cells *in vitro* and in nude mice. Cancer Lett.

[76] Fiume L, Vettraino M, Manerba M, Di Stefano G (2011). Inhibition of lactic dehydrogenase as a way to increase the anti-proliferative effect of multi-targeted kinase inhibitors. Pharmacol Res.

[77] Faloppi L, Bianconi M, Giampieri R, Sobrero A, Labianca R, Ferrari D, Barni S, Aitini E, Zaniboni A, Boni C, Caprioni F, Mosconi S, Fanello S (2015). The value of lactate dehydrogenase serum levels as a prognostic and predictive factor for advanced pancreatic cancer patients receiving sorafenib. Oncotarget.

[78] Jenkinson C, Elliott VL, Evans A, Oldfield L, Jenkins RE, O’Brien DP, Apostolidou S, Gentry-Maharaj A, Fourkala EO, Jacobs IJ, Menon U, Cox T, Campbell F (2016). Decreased Serum Thrombospondin-1 Levels in Pancreatic Cancer Patients Up to 24 Months Prior to Clinical Diagnosis: Association with Diabetes Mellitus. Clin Cancer Res.

[79] Kim J, Bamlet WR, Oberg AL, Chaffee KG, Donahue G, Cao XJ, Chari S, Garcia BA, Petersen GM, Zaret KS (2017). Detection of early pancreatic ductal adenocarcinoma with thrombospondin-2 and CA19-9 blood markers. Sci Transl Med.

[80] Ludwig AK, Giebel B (2012). Exosomes: small vesicles participating in intercellular communication. Int J Biochem Cell Biol.

[81] Lu L, Risch HA (2016). Exosomes: potential for early detection in pancreatic cancer. Future Oncol.

[82] Madhavan B, Yue S, Galli U, Rana S, Gross W, Muller M, Giese NA, Kalthoff H, Becker T, Buchler MW, Zoller M (2015). Combined evaluation of a panel of protein and miRNA serum-exosome biomarkers for pancreatic cancer diagnosis increases sensitivity and specificity. Int J Cancer.

[83] Melo SA, Luecke LB, Kahlert C, Fernandez AF, Gammon ST, Kaye J, LeBleu VS, Mittendorf EA, Weitz J, Rahbari N, Reissfelder C, Pilarsky C, Fraga MF (2015). Glypican-1 identifies cancer exosomes and detects early pancreatic cancer. Nature.

[84] Zhang Y, Yang J, Li H, Wu Y, Zhang H, Chen W (2015). Tumor markers CA19-9, CA242 and CEA in the diagnosis of pancreatic cancer: a meta-analysis. Int J Clin Exp Med.

[85] Gu YL, Lan C, Pei H, Yang SN, Liu YF, Xiao LL (2015). Applicative Value of Serum CA19-9, CEA, CA125 and CA242 in Diagnosis and Prognosis for Patients with Pancreatic Cancer Treated by Concurrent Chemoradiotherapy. Asian Pac J Cancer Prev.

[86] Chan A, Prassas I, Dimitromanolakis A, Brand RE, Serra S, Diamandis EP, Blasutig IM (2014). Validation of biomarkers that complement CA19.9 in detecting early pancreatic cancer. Clin Cancer Res.

[87] Cai CY, Zhai LL, Wu Y, Tang ZG (2015). Expression and clinical value of peroxiredoxin-1 in patients with pancreatic cancer. Eur J Surg Oncol.

[88] Ferri MJ, Saez M, Figueras J, Fort E, Sabat M, Lopez-Ben S, de Llorens R, Aleixandre RN, Peracaula R (2016). Improved Pancreatic Adenocarcinoma Diagnosis in Jaundiced and Non-Jaundiced Pancreatic Adenocarcinoma Patients through the Combination of Routine Clinical Markers Associated to Pancreatic Adenocarcinoma Pathophysiology. PLoS One.

[89] Kaur S, Smith LM, Patel A, Menning M, Watley DC, Malik SS, Krishn SR, Mallya K, Aithal A, Sasson AR, Johansson SL, Jain M, Singh S (2017). A Combination of MUC5AC and CA19-9 Improves the Diagnosis of Pancreatic Cancer: A Multicenter Study. Am J Gastroenterol.

[90] Balasenthil S, Huang Y, Liu S, Marsh T, Chen J, Stass SA, KuKuruga D, Brand R, Chen N, Frazier ML, Jack Lee J, Srivastava S, Sen S (2017). A Plasma Biomarker Panel to Identify Surgically Resectable Early-Stage Pancreatic Cancer. J Natl Cancer Inst.

[91] Capello M, Bantis LE, Scelo G, Zhao Y, Li P, Dhillon DS, Patel NJ, Kundnani DL, Wang H, Abbruzzese JL, Maitra A, Tempero MA, Brand R (2017). Sequential Validation of Blood-Based Protein Biomarker Candidates for Early-Stage Pancreatic Cancer. J Natl Cancer Inst.

[92] Yoneyama T, Ohtsuki S, Honda K, Kobayashi M, Iwasaki M, Uchida Y, Okusaka T, Nakamori S, Shimahara M, Ueno T, Tsuchida A, Sata N, Ioka T (2016). Identification of IGFBP2 and IGFBP3 As Compensatory Biomarkers for CA19-9 in Early-Stage Pancreatic Cancer Using a Combination of Antibody-Based and LC-MS/MS-Based Proteomics. PLoS One.

[93] Kim Y, Kang M, Han D, Kim H, Lee K, Kim SW, Park T, Jang JY (2016). Biomarker Development for Intraductal Papillary Mucinous Neoplasms Using Multiple Reaction Monitoring Mass Spectrometry. J Proteome Res.

[94] Vargas AJ, Harris CC (2016). Biomarker development in the precision medicine era: lung cancer as a case study. Nat Rev Cancer.

[95] Cohen JD, Javed AA, Thoburn C, Wong F, Tie J, Gibbs P, Schmidt CM, Yip-Schneider MT, Allen PJ, Schattner M, Brand RE, Singhi AD, Petersen GM (2017). Combined circulating tumor DNA and protein biomarker-based liquid biopsy for the earlier detection of pancreatic cancers. Proc Natl Acad Sci U S A.

[96] Tempero MA, Uchida E, Takasaki H, Burnett DA, Steplewski Z, Pour PM (1987). Relationship of carbohydrate antigen 19-9 and Lewis antigens in pancreatic cancer. Cancer Res.

[97] Lin C, Wu WC, Zhao GC, Wang DS, Lou WH, Jin DY (2016). ITRAQ-based quantitative proteomics reveals apolipoprotein A-I and transferrin as potential serum markers in CA19-9 negative pancreatic ductal adenocarcinoma. Medicine (Baltimore).

[98] Honda K, Okusaka T, Felix K, Nakamori S, Sata N, Nagai H, Ioka T, Tsuchida A, Shimahara T, Shimahara M, Yasunami Y, Kuwabara H, Sakuma T (2012). Altered plasma apolipoprotein modifications in patients with pancreatic cancer: protein characterization and multi-institutional validation. PLoS One.

[99] Honda K, Kobayashi M, Okusaka T, Rinaudo JA, Huang Y, Marsh T, Sanada M, Sasajima Y, Nakamori S, Shimahara M, Ueno T, Tsuchida A, Sata N (2015). Plasma biomarker for detection of early stage pancreatic cancer and risk factors for pancreatic malignancy using antibodies for apolipoprotein-AII isoforms. Sci Rep.

[100] Jenkinson C, Earl J, Ghaneh P, Halloran C, Carrato A, Greenhalf W, Neoptolemos J, Costello E (2015). Biomarkers for early diagnosis of pancreatic cancer. Expert Rev Gastroenterol Hepatol.

[101] Radon TP, Massat NJ, Jones R, Alrawashdeh W, Dumartin L, Ennis D, Duffy SW, Kocher HM, Pereira SP, Guarner posthumous L, Murta-Nascimento C, Real FX, Malats N (2015). Identification of a Three-Biomarker Panel in Urine for Early Detection of Pancreatic Adenocarcinoma. Clin Cancer Res.

[102] Durczynski A, Kumor A, Hogendorf P, Szymanski D, Grzelak P, Strzelczyk J (2014). Preoperative high level of D-dimers predicts unresectability of pancreatic head cancer. World J Gastroenterol.

[103] Rocker JM, Tan MC, Thompson LW, Contreras CM, DiPalma JA, Pannell LK (2016). Comparative Proteomic Analysis of Whole-Gut Lavage Fluid and Pancreatic Juice Reveals a Less Invasive Method of Sampling Pancreatic Secretions. Clin Transl Gastroenterol.

[104] Majumder S, Chari ST, Ahlquist DA (2015). Molecular detection of pancreatic neoplasia: Current status and future promise. World J Gastroenterol.

[105] Porterfield M, Zhao P, Han H, Cunningham J, Aoki K, Von Hoff DD, Demeure MJ, Pierce JM, Tiemeyer M, Wells L (2014). Discrimination between adenocarcinoma and normal pancreatic ductal fluid by proteomic and glycomic analysis. J Proteome Res.

[106] Chen KT, Kim PD, Jones KA, Devarajan K, Patel BB, Hoffman JP, Ehya H, Huang M, Watson JC, Tokar JL, Yeung AT (2014). Potential prognostic biomarkers of pancreatic cancer. Pancreas.

[107] Matsumoto K, Takeda Y, Harada K, Onoyama T, Kawata S, Horie Y, Sakamoto T, Ueki M, Miura N, Murawaki Y (2015). Clinical Impact of the KL-6 Concentration of Pancreatic Juice for Diagnosing Pancreatic Masses. Biomed Res Int.

[108] Marchegiani G, Paulo JA, Sahora K, Fernandez-Del Castillo C (2015). The proteome of postsurgical pancreatic juice. Pancreas.

[109] Pan S, Brentnall TA, Chen R (2015). Proteomics analysis of bodily fluids in pancreatic cancer. Proteomics.

[110] Singh H, McGrath K, Singhi AD (2017). Novel Biomarkers for Pancreatic Cysts. Dig Dis Sci.

[111] Park J, Yun HS, Lee KH, Lee KT, Lee JK, Lee SY (2015). Discovery and Validation of Biomarkers That Distinguish Mucinous and Nonmucinous Pancreatic Cysts. Cancer Res.

[112] Cuoghi A, Farina A, Z’Graggen K, Dumonceau JM, Tomasi A, Hochstrasser DF, Genevay M, Lescuyer P, Frossard JL (2011). Role of proteomics to differentiate between benign and potentially malignant pancreatic cysts. J Proteome Res.

[113] Streitz JM, Madden MT, Salo W, Bernadino KP, Deutsch JL, Deutsch JC (2014). Differentiation of mucinous from non-mucinous pancreatic cyst fluid using dual-stained, 1 dimensional polyacrylamide gel electrophoresis. Clin Proteomics.

[114] Jabbar KS, Verbeke C, Hyltander AG, Sjovall H, Hansson GC, Sadik R (2014). Proteomic mucin profiling for the identification of cystic precursors of pancreatic cancer. J Natl Cancer Inst.

[115] Jabbar KS, Arike L, Verbeke CS, Sadik R, Hansson GC (2018). Highly Accurate Identification of Cystic Precursor Lesions of Pancreatic Cancer Through Targeted Mass Spectrometry: A Phase IIc Diagnostic Study. J Clin Oncol.

[116] Navaneethan U, Lourdusamy V, Gk Venkatesh P, Willard B, Sanaka MR, Parsi MA (2015). Bile proteomics for differentiation of malignant from benign biliary strictures: a pilot study. Gastroenterol Rep (Oxf).

[117] Terai K, Jiang M, Tokuyama W, Murano T, Takada N, Fujimura K, Ebinuma H, Kishimoto T, Hiruta N, Schneider WJ, Bujo H (2016). Levels of soluble LR11/SorLA are highly increased in the bile of patients with biliary tract and pancreatic cancers. Clin Chim Acta.

